# A new amyloid PET evaluation method using separated gray-matter histogram based on three-component model

**DOI:** 10.1007/s12149-026-02157-5

**Published:** 2026-02-07

**Authors:** Chio Okuyama, Naoya Oishi, Koichi Ishizu, Hiroshi Hasegawa, Miki Ito, Yoshiharu Fujita, Kuninori Kusano, Tomoko Okina, Shinya Kagawa, Hiroyuki Watanabe, Tatsuya Higashi, Hiroshi Yamauchi, Masahiro Ono

**Affiliations:** 1 Clinical Research Center , Shiga General Hospital , Moriyama, Japan; 2 Human Brain Research Center Graduate School of Medicine , Kyoto University , Kyoto, Japan; 3 Graduate School of Medicine , Human Health Sciences Kyoto University , Kyoto, Japan; 4 Department of Neurology , Shiga General Hospital , Moriyama, Japan; 5 Department of Radiology , Shiga General Hospital , Moriyama, Japan; 6 Department of Patho-Functional Bioanalysis Graduate School of Pharmaceutical Sciences , Kyoto University , Kyoto, Japan; 7 Department of Psychiatry Graduate School of Medicine , Kyoto University , Kyoto, Japan; 8 Department of Molecular Imaging and Theranostics Institute for Quantum Medical Sciences , National Institutes for Quantum Science and Technology , Chiba, Japan

**Keywords:** Amyloid PET, Histogram, Gray matter, Three-component model, Objective evaluation, F-18-FPYBF-2

## Abstract

**Objective:**

We have constructed a three-component model underlying amyloid PET accumulation and developed a new gray matter histogram evaluation method based on this model. This study aims to validate the utility of the new method compared with conventional visual and SUVR-based quantitative evaluation.

**Methods:**

A retrospective analysis was performed on amyloid PET/CT data from 63 participants (25 healthy volunteers, 38 patients with dementia or cognitive impairment) of previous study using ^18^F-FPYBF-2. Subjects were visually classified into three groups: negative, borderline, and positive, and quantitatively evaluated using composed standardized uptake value (comSUVR) with a reference to cerebellar cortex. Histograms were generated for the whole-brain, gray matter (GM-histogram), and white matter (WM-histogram) based on probability-tissue maps. The GM-histogram was further decomposed into two Gaussian components: G1 and G2　using statistical software. Parameters of whole-brain histogram: skewness, mode-to-mean ratio (MMR), and parameters of GM-histogram: GM-kurtosis, µG2 (mean of G2), and πG2 (proportion of G2), were compared among visual groups and the correlation with comSUVR was evaluated.

**Results:**

The GM-histogram was sharply unimodal in visually negative group but showed a wide shape to bimodal patterns in visually positive cases. Visually border group showed significantly higher πG2 than negative group, and positive group showed significantly higher µG2 than border group. GM-kurtosis and µG2 showed stronger negative (*p* < 0.0001, R^2^ = 0.7539) and positive (*p* < 0.0001, R^2^ = 0.8589) correlations with ComSUVR, respectively than the correlations between whole-brain histogram parameters and ComSUVR.

**Conclusion:**

Our proposed GM-histogram provides a visually comprehensive morphology and quantitative indicators that match conventional visual and SUVR-based assessments and may potentially detect even subtle amyloid accumulation. This method is considered promising as a complementary tool for early diagnosis and treatment monitoring of Alzheimer’s disease.

## Introduction

Extracellular amyloid beta (Aβ) deposition is known the first step of the neuropathological change of Alzheimer’s disease (AD) [1–3]. On January 6, 2023, lecanemab, humanized monoclonal antibody targeting soluble Aβ protofibrils, was approved by U.S. Food and Drug Administration for patients with mild cognitive impairment (MCI) and early dementia caused by AD. On July 2, 2024, donanemab targeting directedly against an insoluble form of Aβ was also approved [4]. Amyloid positron emission tomography (PET), which shows a strong correlation with the intracranial Aβ distribution [5], thus, has now become the requisite imaging tool for diagnosing, determining treatment options for patients, and the evaluation of the response to donanemab when determining whether to continue the treatment [6].

Pittsburgh compound B (PiB) labeled with ^11^C, the first tracer for Aβ, was developed in 2003 [7] and has been used for research for years. The short half-life of ^11^C (20 min), though, limits its applicability for clinical practice [8], and several ^18^F-labeled tracers have been developed to overcome the drawback of ^11^C-PiB [9–11]. 5-(5-(2-(2-(2-^18^ F-fluoroethoxy) ethoxy) ethoxy) benzofuran-2-yl)-N-methylpyridin-2-amine (^18^F-FPYBF-2) is one of the new ^18^F-labeled amyloid tracers which our coauthors developed in 2011 [12]. So far, using this tracer, we published the first-in-human data of the biodistribution and radiation dosimetry and its safety [13],　and its clinical utility to evaluate AD and related diseases which showed its comparable diagnostic ability with ^11^C-PiB [14].

Each tracer has its unique intracranial physiological distribution, but whichever tracer is used, visual evaluation is considered essential. How to evaluate, or what color scales to use are determined in detail for each radiopharmaceutical [15], but the discrepancies in the interpretation between readers are unavoidable [16]. Quantitative evaluation with standardized uptake value ratio (SUVR), the relative accumulation ratio with some reference sites, is also used [17, 18]. In this decade, Centiloid scale, a numerical parameter based on SUVR evaluation to grade the amyloid accumulation with a scale from 0 indicating no amyloid deposition to 100 indicating definitive AD [19], is widely used as a common index on amyloid deposition for research and clinical practice. For such evaluation, manually placing region of interests (ROIs) on the cerebral cortex is less reproducible and requires strenuous work; therefore, ROI-templates which are spatially normalized into standard Montreal Neurological Institute’s(MNI) space [20] are often applied after converting to the standard brain. The problem of quantitative inaccuracies involved in these methods is well recognized [21]. Moreover, the cerebral cortex, the target area for amyloid PET, has a tortuous shape adjacent to the white matter with high physiological tracer accumulation underneath. Considering the resolution of PET, it is not easy to evaluate the ROI of the cortex with only a-few-millimeters thickness [22] without being influenced by the white matter component.

**Fig. 1:**
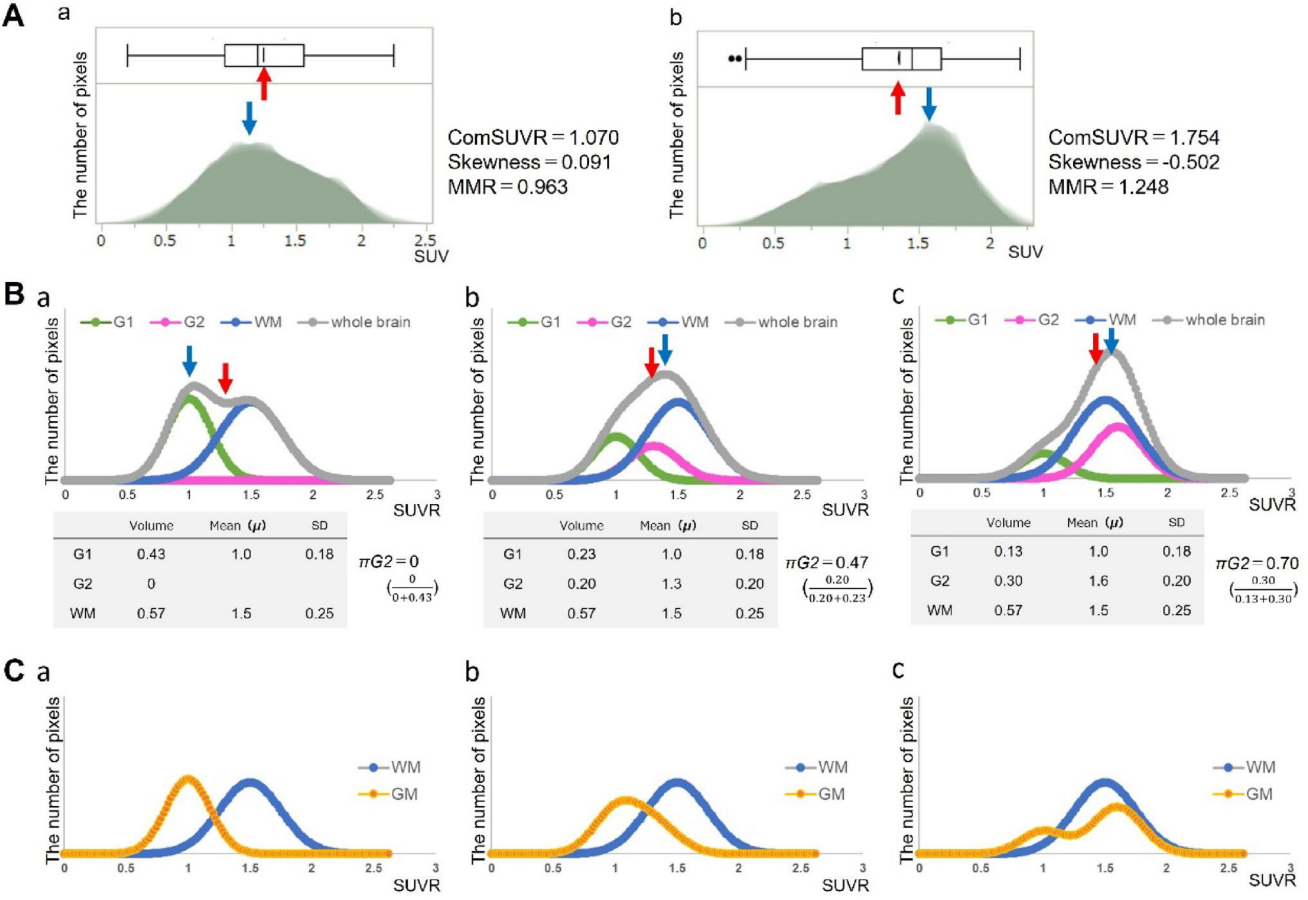
Three-component model showing the formation of histograms A: Whole-brain histograms of a healthy individual (**a**), and a patient with Alzheimer’s disease (**b**). (Cited from the authors’ previous publication [23]) MMR: mode to mean ratio B: The histograms created with three-component-model hypothesis of three case models: a, without abnormal amyloid accumulation; b, with mild abnormal amyloid accumulation; c, with large amount of abnormal amyloid accumulation C: GM-histograms and WM-histograms of the same three case models as in B red arrow, mean value; blue arrow, mode value

Recently, we have developed a simple objective amyloid PET evaluation method using histogram of the SUV data without any reference site in the whole brain parenchyma　(Fig. 1A) [23]. This method is quite different from conventional ROI analyses but only uses individuals’ brain data. The whole-brain histograms of healthy subjects have a peak to the left (low accumulation) side of the center (a), while those of cases with AD exhibit a peak on the right side (high accumulation) (b). The skewness, and mode to mean ratio (MMR), the parameters obtained from the whole-brain histogram, showed significantly good negative and positive correlation with SUVR, respectively. Whole-brain histogram, thus, provides visually comprehensive information about intracranial pathological amyloid deposition, and the similarity in the evaluation using these parameters between ^11^C-PiB and ^18^F-FPYBF-2 was also confirmed [23].

To elucidate the mechanism forming such whole-brain histograms we herein propose a three-component-model hypothesis. Figure 1B shows an overview of the histogram morphologies of 3 case-models with following different amounts of pathological amyloid deposition: a, non; b, mild; c, severe. This hypothesizes that the brain parenchyma comprises three components: G1, gray matter without pathological amyloid deposition; G2, gray matter with pathological amyloid deposition; WM, white matter rich in the off-target physiological tracer uptake, and the amyloid PET tracer accumulation of each component follows a Gaussian distribution. The tracer uptake is expressed using SUVR with cerebellar cortex as the reference site. The green, pink, blue lines shown in Fig. 1B are the histograms of SUVR of G1, G2, and WM components, respectively, created using a general spreadsheet software Excel (Microsoft 365) by specifying the mean value the Gaussian distribution (*µG1*, *µG2*, and *µW* respectively), volume fraction, and standard deviation shown in the table below the figures. The mean value, volume fraction, and standard deviation are shown in the table below the figures. The another parameter　“*πG2*” represents the proportion of the volume occupied by the G2 component in the gray matter. The silver lines show the whole-brain histograms constructed as the summation of the three components.

The ratios of the gray and white matter volumes to the total brain change with age or various diseases [24–26], and they are reported to be approximately 40–45% and 55–60%, respectively [27–30]. Our hypothetical model, therefore, assumed them to be 43% and 57%, respectively. In addition, we set *µG1* = 1, assuming that the tracer accumulation in the gray matter without pathological amyloid deposition is comparable in the cerebellar cortex, and *µWM* = 1.5, since off-target accumulation in the white matter is higher than the cerebellar cortex.

The shape of the whole-brain histogram of the hypothetical model changes depending on the proportion and extent of the G2 component (Fig. 1B), similar to the actual whole-brain histograms shown in our previous study (Fig. 1A).

Figure 1C shows the separated histograms, the summation of G1 and G2 (GM-histogram: orange line), and white matter histogram (WM-histogram: blue line) of the same case models. The GM-histogram shows one narrow peak (Fig. 1C-a) in the model with little amyloid deposition, and as amyloid deposition increases, it gradually becomes a wide peak (Fig. 1C-b) then two-peaks form (Fig. 1C-c).

Based on this hypothetical model, we developed another histogram analysis that separates gray matter and white matter using probabilistic tissue maps (https://fsl.fmrib.ox.ac.uk/fsl/fslwiki/Atlases). The purpose of this study was to investigate the utility of the separated GM-histogram in a retrospective manner compared with conventional methods, using the same ^18^F-FPYBF-2 PET/CT data as our prior study [23].

## Materials and methods

This study was approved by our institutional review boards, the Human Study Committee (approved on Sep༎7th, 2021) and was performed in accordance with the Ethical Guidelines for Medical and Biological Research Involving Human Subjects by Japanese Ministry of Health, Labor and Welfare. This study was partially supported by a grant from Grants-in-Aid for Scientific Research KAKENHI (JSPS Grant Number JP21K07635).

### Subjects

^18^F-FPYBF-2 PET/CT data were obtained in our previous study [14]: the first-in-human study using ^18^F-FPYBF-2 for subjects with cognitive disorders or healthy volunteers (HV), and the comparative study between ^18^F-FPYBF-2 and ^11^C-PiB PET, which were approved by our institutional review boards, the Human Study Committee (approved on Sep. 25, 2013, and on Jan. 18, 2016). The current analyses included the same 63 participants (25 HV and 38 patients, 27 men and 36 women; mean age: 68.7 ± 12.4 years old) as our prior whole-brain histogram analysis [23]. The patient group included 15 patients clinically diagnosed with AD, 17 with MCI, and 6 with other conditions (e.g., dementia of Lewy bodies, cerebral amyloid angiopathy and vascular dementia, and front-temporal lobe dementia (Others)). Written informed consent was obtained for the previous studies. Due to the retrospective study design and use of anonymized data, the requirement for additional consent was waved.

## PET/CT examination

^18^F-FPYBF-2 was synthesized in-house using modification of the methods described elsewhere [13]. A static brain PET/CT scan was acquired from 50 to 70 min after intravenous administration of the tracer (3.7–4.7.0MBq/kg) using TruePoint Biograph 16 (Siemens/CTI, Erlangen, Germany). Acquired PET data with a 256 × 256 matrix were reconstructed using the back projection, and images were blurred to 6.0 mm full width at half maximum in the trans-axial direction using a Gaussian filter. CT images performed for attenuation correction were resized from 336 × 336 to 256 × 256 matrices to match the PET images, and processed with a 5 mm FWHM Gaussian post-filter. The final PET/CT voxel size was 2.3 × 2.3 × 4.0 mm.

## The creation of SUVR images and histogram

For quantitative analysis, PET images were spatially normalized to standard brain space and automated ROI analysis was performed using the automated anatomical labeling (AAL) atlas [31] as described in our previous study [14]. The ROIs on the cerebellar cortex were combined and used as a reference region and we calculated regional SUVR (rSUVR) for each ROI [32] by an in-house Matlab script. Then, as a value for abnormal cortical amyloid deposition of each subject, the mean rSUVR value within the frontal, posterior cingulate, precuneus, parietal and lateral temporal cortical regions was calculated as composed SUVR (ComSUVR).

**Fig. 2:**
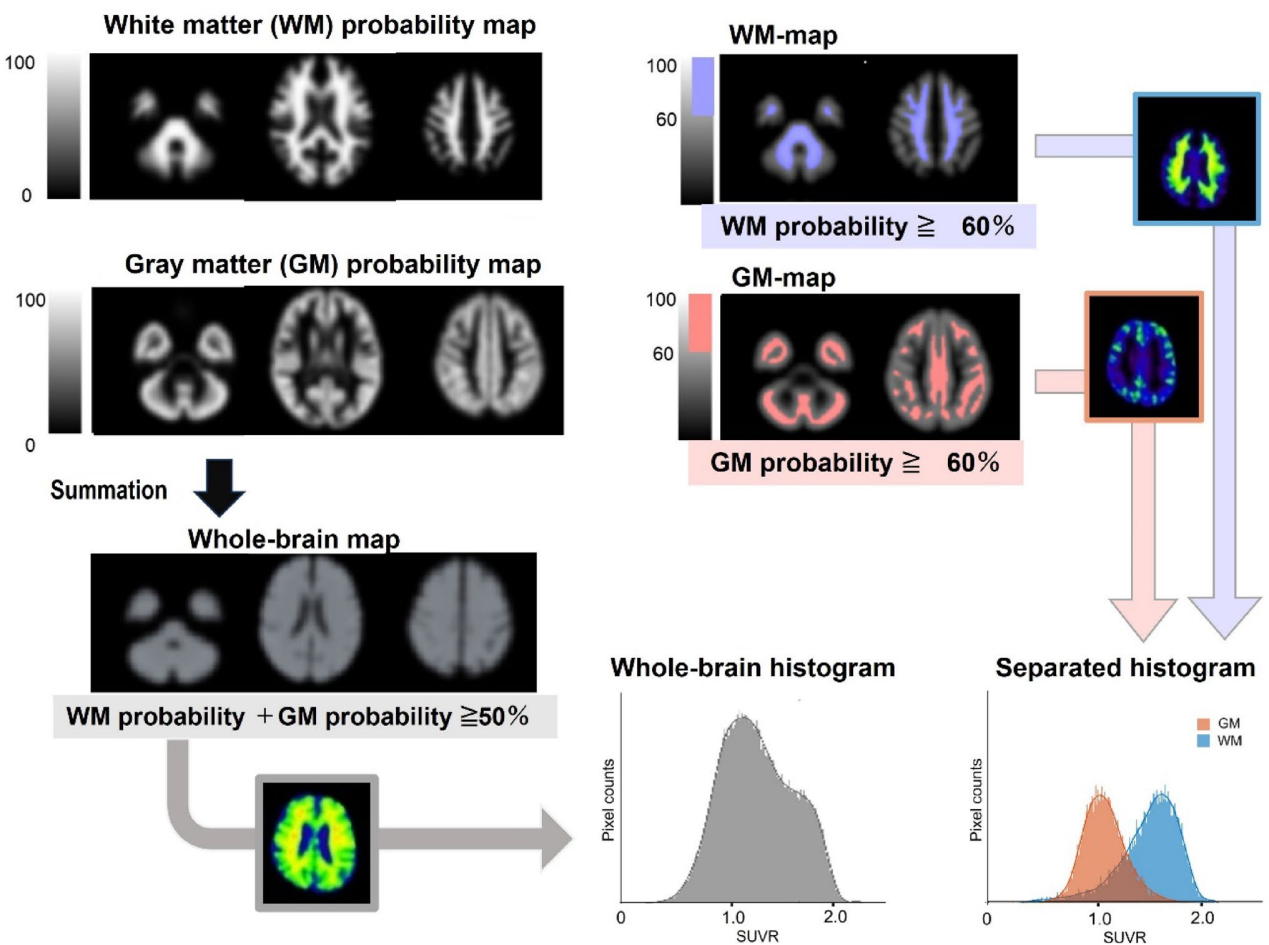
The process to create the separated histograms

Figure 2 demonstrates the scheme of the steps of the histogram creation. To separate individual brain data into gray and white matter and create histograms, we used publicly available probabilistic tissue maps (https://fsl.fmrib.ox.ac.uk/fsl/fslwiki/Atlases), which was developed by Oxford Centre for Functional MRI of the Brain to automatically classify brain tissue for various brain analyses, such as quantifying brain atrophy or analyzing brain structural changes associated with development and aging [37]. Prior to the histogram creation, individual PET images on SUVR and probabilistic tissue maps were up-sampled using bicubic interpolation to enhance histogram resolution and reduce discretization artifacts.

The whole-brain histogram was constructed from SUVR values within brain parenchyma, excluding the cerebrospinal fluid space. Specifically, only voxels where the sum of gray matter (GM) and white matter (WM) probabilities exceeded 50% were included, to ensure that the analysis was restricted to voxels within the brain tissue. In addition, tissue-specific separated histograms, GM-histogram and WM-histogram, were generated. For the GM-histogram, only voxels with GM probability greater than 60% (GM-map), were included, and likewise, the WM-histogram was constructed using voxels with WM probability greater than 60% (WM-map). This approach excluding the boundaries between GM and WM differs from our original hypothetical model in which all pixels of the brain parenchyma are classified into one of three components, but was intended to reduce partial volume effects and to minimize the influence of non-specific high accumulation in the white matter on the GM-histogram.

## The data analysis

### Classification by visual evaluation

The axial, coronal, and sagittal PET images were evaluated using rainbow color and gray scale expressed with the SUVR display (range: 0–3). For the gray scale images, we also evaluated by freely adjusting the gradation of the DICOM image on the image viewer. As described in the prior study [23], the PET images were visually evaluated by three experienced reviewers with 3 points (0: negative, 1: equivocal, and 2: positive). According to the score in which the three reviewers’ points were summed up, the 63 subjects were classified into 32 negative (summed score = 0 or 1), 10 border cases (summed score = 2–4), and 21 positive (summed score = 5 or 6), subjects. For the purpose to validate our new evaluation methods, the results of visual evaluation were used as a reference regardless of the clinical diagnosis that is sometimes corrected after amyloid PET.

### Histogram analyses

Histogram data with the bin width of SUVR 0.05, were analyzed using JMP^®^15.3.0 statistical software (SAS Institute Japan, Tokyo). In order to distinguish from the parameters shown in *italic* font of the hypothetical model described in the introduction section, the parameters obtained from actual PET data are shown in non-italic font. From the whole-brain histogram data, the parameters including skewness, mean, and mode values were extracted. Skewness was calculated using the following formula:

$$\:\mathrm{S}\mathrm{k}\mathrm{e}\mathrm{w}\mathrm{n}\mathrm{e}\mathrm{s}\mathrm{s}=\frac{\mathrm{n}}{(\mathrm{n}-1)(\mathrm{n}-2)}\sum\:_{\mathrm{i}=1}^{\mathrm{n}}{\left(\frac{{\mathrm{x}}^{\mathrm{i}}-{\mathrm{x}}^{\mathrm{m}}}{\mathrm{s}}\right)}^{3}$$ $$\:\:{\mathrm{x}}_{\mathrm{m}},\:\mathrm{m}\mathrm{e}\mathrm{a}\mathrm{n}\:\mathrm{v}\mathrm{a}\mathrm{l}\mathrm{u}\mathrm{e};\:\mathrm{s},\:\mathrm{s}\mathrm{t}\mathrm{a}\mathrm{n}\mathrm{d}\mathrm{a}\mathrm{r}\mathrm{d}\:\mathrm{d}\mathrm{e}\mathrm{v}\mathrm{i}\mathrm{a}\mathrm{t}\mathrm{i}\mathrm{o}$$n.

Then mode to mean ratio (MMR) was calculated according to the following formula: $\:\mathrm{M}\mathrm{M}\mathrm{R}=\mathrm{m}\mathrm{o}\mathrm{d}\mathrm{e}\:\mathrm{v}\mathrm{a}\mathrm{l}\mathrm{u}\mathrm{e}\:/\mathrm{m}\mathrm{e}\mathrm{a}\mathrm{n}\:\mathrm{v}\mathrm{a}\mathrm{l}\mathrm{u}\mathrm{e}$

From the data of GM-histogram, its kurtosis (GM-kurtosis) was calculated using the following formula:

$$\:\mathrm{K}\mathrm{u}\mathrm{r}\mathrm{t}\mathrm{o}\mathrm{s}\mathrm{i}\mathrm{s}=\frac{\mathrm{n}(\mathrm{n}+1)}{(\mathrm{n}-1)(\mathrm{n}-2)(\mathrm{n}-3)}\sum\:_{\mathrm{i}=1}^{\mathrm{n}}\frac{{({\mathrm{x}}_{\mathrm{i}}-{\mathrm{x}}_{\mathrm{m}})}^{4}}{{\mathrm{S}}^{4}}-\frac{3(\mathrm{n}-1)}{(\mathrm{n}-2)(\mathrm{n}-3)}$$.

$$\:\:{\mathrm{x}}_{\mathrm{m}},\:\mathrm{m}\mathrm{e}\mathrm{a}\mathrm{n}\:\mathrm{v}\mathrm{a}\mathrm{l}\mathrm{u}\mathrm{e};\:\mathrm{s},\:\mathrm{s}\mathrm{t}\mathrm{a}\mathrm{n}\mathrm{d}\mathrm{a}\mathrm{r}\mathrm{d}\:\mathrm{d}\mathrm{e}\mathrm{v}\mathrm{i}\mathrm{a}\mathrm{t}\mathrm{i}\mathrm{o}\mathrm{n}$$.

In the next step, GM-histogram was separated into two Gaussian distributions using JMP^®^15.3.0. The mean value of each Gaussian distribution was defined as µG1 and µG2, respectively (µG1 < µG2). The proportion of number of the pixels in the GM that belong to G2 is defined as πG2. From the WM-histogram, µW　was also calculated.

For each parameter obtained from the histogram, a comparison was made among the three groups divided by visual evaluation, and the correlation with ComSUVR was evaluated. If a significant age difference among groups was observed based on visual assessment, age-matched data were used only when examining group comparisons in order to eliminate the influence of age.

### Statistical analysis

Statistical analyses were performed using JMP>^®^15.3.0 with p values < 0.05 denoting statistical significance. Comparisons among three groups were analyzed by Kruskal-Wallis tests. If statistically significant differences were observed among the three groups, comparisons were then made between each group. Spearman’s rank tests were performed for the correlation between the new parameters obtained histograms and ComSUVR.

## Results

### Numerical data according to the visual groups

Table 1 shows the results of the numerical data according to the three visual evaluation groups. A　significant age difference was observed among the three groups because the visually assessed negative group included a large number of healthy volunteers. Therefore, in order to compare each parameter among the three groups, we examined age-matched data by excluding 15 younger healthy volunteers from the visually assessed negative group.　There were significant differences in ComSUVR, Skewness, MMR, GM-kurtosis, µG2 (*p* < 0.0001) and πG2 (*p* < 0.001). In the intergroup comparison, µG2 of visually positive group was significantly higher but negative and border group showed no statistically significant difference. On the contrary, πG2 differed significantly between the border and negative groups. No significant differences were observed in µG1 and µW among the groups. The µG1 values ​​for all groups showed little variation and were approximately 1.0, while there was wide variation in the distribution of µW values in each group.

**Table 1:** The summary of the numerical data according to the visual classification

		Visual evaluation			*P* values				
		**Negative (N)** (*n* = 32)	**Border (B)** (*n* = 9)	**Positive (P)** (*n* = 22)		Inter-group comparison			
		(*n* = 32)	(*n* = 9)	(*n* = 22)		*N* vs. B	B vs. *P*	*N* vs. *P*	
**Clinical Diagnosis before PET**									
	HV	23*	1	1					
	AD	1	2	12					
	MCI	6	5	6					
	Others	2	1	3					
**Age**		63.3 ± 14.0 (60 [52,78])	73.1 ± 8.6 (73 [68, 81.5])	74.6 ± 6.9 (77 [60.5, 79])	< 0.0001	n.s.	n.s.	< 0.0001	
Comparison among the groups classified by visual evaluation using age-matched subjects*									
		**Negative (N)** (*n* = 17)	**Border (B)** (*n* = 9)	**Positive (P)** (*n* = 22)	P value	N vs. B	B vs. P	N vs. P	
**Age**		71.9 ± 11.1 (72 [67, 78.5])	73.1 ± 8.6 (73 [68, 81.5])	74.6 ± 6.9 (77 [60.5, 79])	n.s.				
**ComSUVR**		1.12 ± 0.07 (1.14 [1.08,1.18])	1.20 ± 0.13 (1.17 [1.12, 1.38])	1.48 ± 0.18 (1.44 [1.36, 1.57])	< 0.0001	n.s.	< 0.005	< 0.0001	
**Whole-brain histogram parameters**									
	Skewness	−0.05 ± 0.17 (0.01 [−0.20, 0.11])	−0.20 ± 0.13 (−0.18 [−0.30, −0.09])	−0.56 ± 0.16 (−0.57 [−0.65, −0.44])	< 0.0001	n.s.	< 0.0005	< 0.0001	
	MMR	0.93 ± 0.05 (0.91 [0.90, 0.97])	1.02 ± 0.06 1.01 [0.96, 1.04])	1.11 ± 0.06 1.12 [1.07, 1.16])	< 0.0001	< 0.005	< 0.001	< 0.0001	
**Separated GM-histogram parameters**									
	GM-kurtosis	0.17 ± 0.21 (0.21 [0.02, 0.35])	−0.15 ± 0.30 (−0.11 [−0.41, 0.08])	−0.59 ± 0.38 (−0.70 [−0.89, −0.42])	< 0.0001	n.s.	< 0.005	< 0.0001	
	µG1	1.04 ± 0.08 (1.04 [1.01, 1.09])	1.03 ± 0.03 (1.04 [0.99, 1.05])	1.06 ± 0.06(1.05 [1.02, 1.09])	n.s.				
	µG2	1.23 ± 0.10 (1.23 [1.19, 1.30])	1.34 ± 0.13 (1.29 [1.22, 1.48])	1.60 ± 0.22 (1.62 [1.43, 1.71])	< 0.0001	n.s.	< 0.005	< 0.0001	
	µW	1.55 ± 0.12 (1.56 [1.50, 1.61])	1.58 ± 0.16 (1.54 [1.48, 1.69])	1.59 ± 0.20 (1.57 [1.45, 1.77])	n.s.				
	πG2	0.35 ± 0.18 (0.34 [0.21, 0.42])	0.48 ± 0.19 (0.51 [0.27, 0.67])	0.54 ± 0.13(0.55 [0.41, 0.62])	< 0.001	< 0.05	n.s.	< 0.005	

Values are expressed as mean ± Standard deviation (median [1st quartile, 3rd quartile]), n.s.: not significant

### Correlation between histogram parameters and comsuvr

**Fig. 3:**
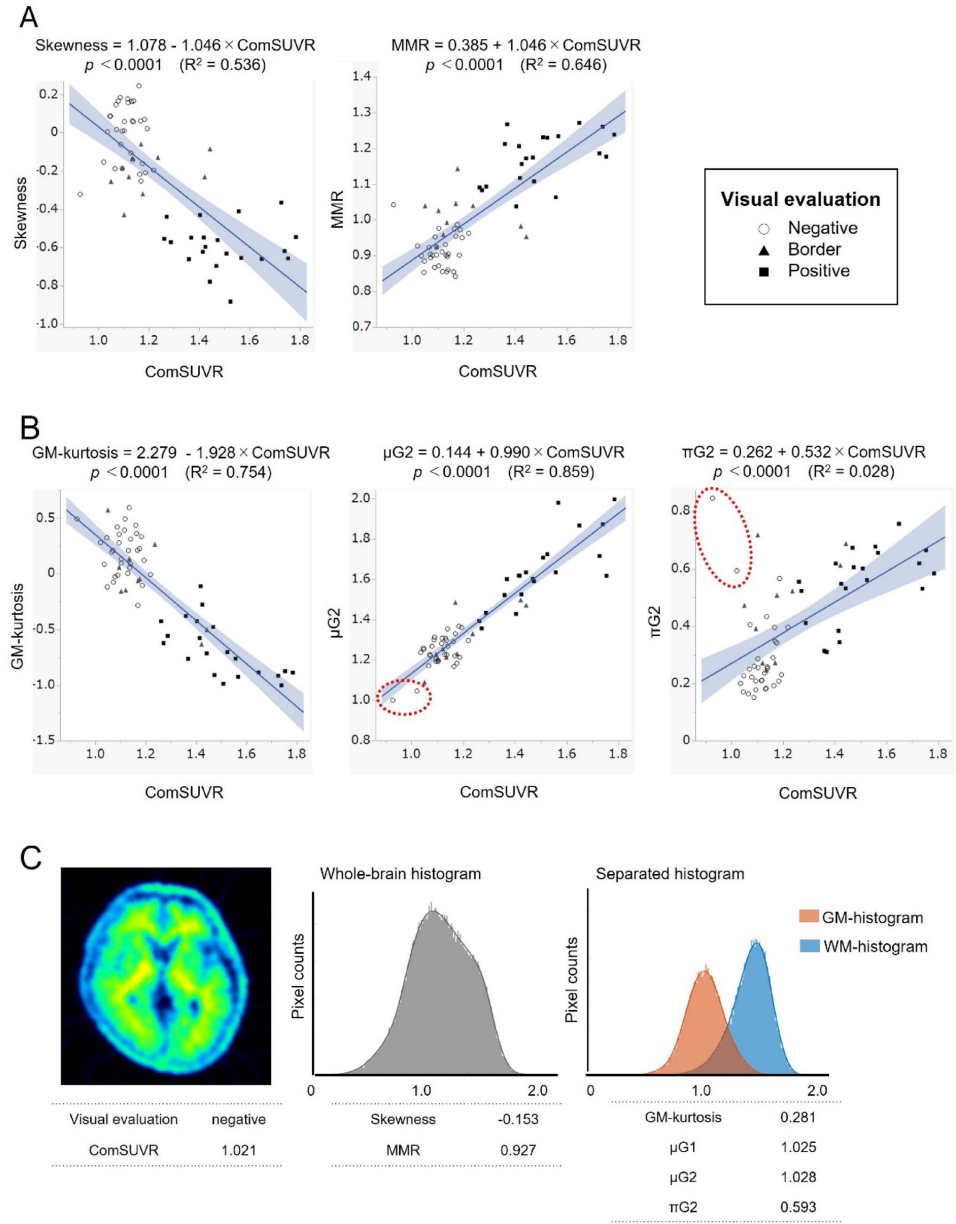
A: Relationships between ComSUVR and the parameters of whole-brain histogram (skewness and MMR) B: Relationships between ComSUVR and GM-histogram (GM-kurtosis, µG2, and πG2). The two visually negative cases circled with dashed lines are clearly outliers (with high πG2 values) in the graph showing the correlation between ComSUVR and πG2. However, these cases also show really low µG2 values close to 1 C: A PET image, histogram, and parameters of one example from the dashed circle in Fig. 3B. A visually negative case in which the G2 component cannot be visually recognized in the GM-histogram. It is presumed that forcibly separating the gray matter into G1 and G2 components with nearly the same µ values through calculation may cause outliers in πG2

Figure 3A shows the correlation between ComSUVR values ​​and whole-brain histogram parameters of the all 63 subjects. The skewness and MMR showed a strong negative (R^2^ = 0.536, *p* < 0.0001) and a strong positive (R^2^ = 0.646, *p* < 0.0001) association with the ComSUVR, respectively.　The values of these parameters from whole-brain histograms are almost similar as those of the prior study [23], but not the same because the ways to extract the brain parenchyma were different.

Figure 3B shows the correlation between ComSUVR values ​​and GM-histogram parameters. GM-kurtosis showed a negative (R^2^ = 0.754 *p* < 0.0001), and µG2 showed a positive (R^2^ = 0.859, *p* < 0.0001) significant and much stronger association with ComSUVR compared with the parameters obtained from whole-brain histograms. On the other hand, the correlation between πG2 and ComSUVR was very weak (R^2^ = 0.028, *p* < 0.0001). This was due to the influence of two visually negative with a unimodal-shape GM-histogram cases with high πG2 and low ComSUVR (surrounded with dashed lines). The PET image and histograms of one of these cases were shown in Fig. 3C. The high πG2 was considered likely because µG2 was so small that it was visually difficult to separate the G1 and G2 components.

### Representative cases presentation

**Fig. 4:**
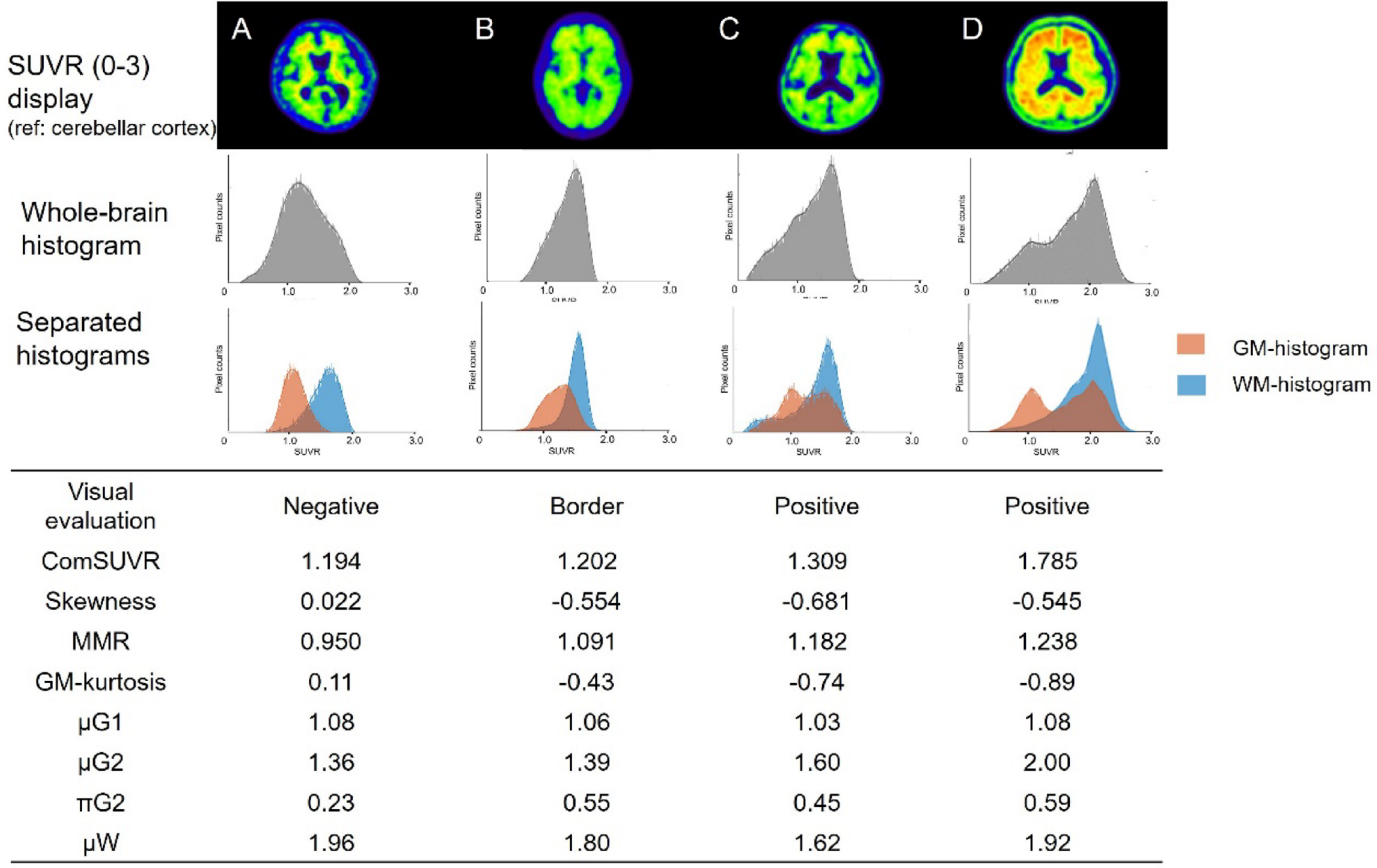
PET Images, whole-brain and separated histograms, and these parameters of representative 4 cases

Figure 4 shows the PET images, whole-brain histograms, separated histograms and numerical values of four representative cases with clinically suspected as having AD before undergoing FPYBF-2 PET/CT. Visually, case A, B, C, and D were judged negative, border, positive, and positive. GM-histogram showed one narrow peak in case A (GM-kurtosis = 0.11), one but wide peak in case B (GM-kurtosis = −0.43), and two peaks in case C and D. Although every case showed almost the same value of µG1, the difference in the values of µG2 and πG2 showed the characteristics of the GM-histogram shapes. µW appeared to be unrelated to accumulation in gray matter.

## Discussion

In this study, we built a three-component model hypothesis that divides the intracranial distribution of amyloid PET tracer into three regions: gray matter without amyloid deposits (G1), gray matter with amyloid deposits (G2), and white matter showing nonspecific accumulation (WM), and developed a new amyloid-PET evaluation method constructing GM- and WM- histograms. Our results confirmed that the shape of newly developed GM-histogram changes depending on the degree of abnormal amyloid accumulation in the brain and validated the three-component model hypothesis (shown in Fig. 4).

Decomposing a GM-histogram into two Gaussian distribution, G1 and G2, we evaluated the following parameters: µG1, µG2, and πG2. As hypothesized, the value of µG1 showed almost 1.0 in all tracer accumulation, while µG2, and πG2 significantly differed depending on amyloid deposition. In visually positive group showed significantly higher µG2 values, and µG2 was strongly correlated with ComSUVR. Though πG2 showed only a weak correlation with ComSUVR, it showed significantly higher values in visually border group than visually negative group. These new parameters from GM-histogram would help to express slight changes that may not be easy to detect with visual assessment or SUVR alone.

On the other hand, there were some differences between the three-component model and the actual data. Even visually negative cases had a G2 component with µG2 of approximately 1.2 to 1.3 and πG2 of approximately 0.3. This small G2 component may reflect a small amount of amyloid deposition or may be influenced by the white matter components included in the GM-map. However, as shown in Fig. 3B, there are some visually negative cases, surrounded by the red dashed lines, whose G2 components almost undistinguishable from G1 component caused µG1 values around 1.0　and extremely high πG2 values. Although πG2 may be able to detect slight accumulations, it should not be judged solely by πG2, but should be evaluated in conjunction with the shape of the histogram.

Regarding µW which we set to 1.5 in the hypothetical model, it showed a wide range of values ​​in clinical cases. Although Lowe et al. reported that white matter accumulation increases with age and is associated with the level of amyloid deposition in gray matter [33], the factors that determine the amount of off-target white matter uptake have not been fully elucidated yet, and our results showed no significant differences among age-matched visual groups.　The large variability of µW among the individuals raises the concern that the conventional visual assessment comparing the degree of accumulation in the gray with that in the white matter, may miss mild amyloid deposition when off-target accumulation in is high. Our new method, which focuses on GM-histogram, can capture the G2 component from two perspectives: an increase in πG2 or in µG2. This method is less affected by the amount of off-target accumulation and may be able to detect mild accumulation in the gray matter, which may be difficult by relative comparison with in the white matter.

Although the amyloid distributes widely in the gray matter in most AD cases, it is known to localize to some specific regions in some cases [34]. In hereditary AD, amyloid deposition often begins in the cerebellum [35] or striatum [36]. Several available software designed for amyloid quantification and Centiloid scale calculation often fails to detect these localized accumulations [37]. Our new method does not completely overcome the shortcomings of these conventional methods, and further studies incorporating a larger dataset are needed to validate the utilities of this method. Furthermore, the true usefulness of the GM-histogram should be further discussed by evaluating changes over time, such as the natural progression of the disease or post-treatment responses.

There are some limitations in this study. First, we used ^18^F-FPYBF-2, which is a less familiar tracer, so its applicability for other tracers needs to be further evaluated.

Second, the appropriateness of setting the probability threshold at 60% when creating the separated histograms has not been fully verified༎If the threshold is set at around 50% probability, both GM-map and WM-map will include many pixels in the boundary area of both matters, making it difficult to separate them properly. On the other hand, setting at a higher probability reduces the number of the pixels resulting in lesser certainty. After several attempts (data not shown), the 60% threshold is considered adequately to depict the characteristics in individual cases. Some other ideal methods for extracting gray matter could provide better histograms.

Third, pathological amyloid deposits of patients with AD are not uniform throughout the brain. In this study, the GM-histogram was separated into two Gaussian distributions using analysis software considering abnormal amyloid deposits as a single component, “G2”, but the complex amyloid deposition process may sometimes be more accurately described by dividing it into three or more components. No matter how many G2 components exist, the shape of the histogram can intuitively serve as a key to indicate their distribution.

In conclusion, the gray-matter histogram we developed is a novel method that can provide auxiliary information, including a visually appealing quantitative assessment of pathological amyloid deposition, and is expected to be applied to the evaluation of pathology and progression in the future.
